# Changes in body mass index during early childhood on school‐age asthma prevalence classified by phenotypes and sex

**DOI:** 10.1111/ped.70090

**Published:** 2025-06-03

**Authors:** Toshihiko Yabuuchi, Masanori Ikeda, Naomi Matsumoto, Mitsuru Tsuge, Takashi Yorifuji, Hirokazu Tsukahara

**Affiliations:** ^1^ Department of Pediatrics, Graduate School of Medicine, Dentistry, and Pharmaceutical Sciences Okayama University Okayama Japan; ^2^ Department of Pediatrics Okayama University Medical School Okayama Japan; ^3^ Department of Epidemiology, Graduate School of Medicine, Dentistry and Pharmaceutical Sciences Okayama University Okayama Japan

**Keywords:** asthma, body mass index, child, phenotypes, sex

## Abstract

**Background:**

Few studies have explored the relationship between changes in body mass index(BMI) during early childhood and asthma prevalence divided by phenotypes and sex, and the limited results are conflicting. This study assessed the impact of BMI changes during early childhood on school‐age asthma, classified by phenotypes and sex, using a nationwide longitudinal survey in Japan.

**Methods:**

From children born in 2001 (*n* = 47,015), we divided participants into BMI quartiles (Q1, Q2, Q3, and Q4) and the following BMI categories: Q1Q1 (i.e., Q1 at birth and Q1 at age 7), Q1Q4, Q4Q1, Q4Q4, and others. Asthma history from ages 7 to 8 was analyzed, with bronchial asthma (BA) further categorized as allergic asthma (AA) or nonallergic asthma (NA) based on the presence of other allergic diseases. Using logistic regression, we estimated the asthma odds ratio (OR) and 95% confidence intervals (CIs) for each BMI category.

**Results:**

Q1Q4 showed significantly higher risks of BA, AA, and NA. In boys, BA and NA risks were significantly higher in Q1Q4 (adjusted OR: 1.47 [95% CI: 1.17–1.85], at 1.56 [95% CI: 1.16–2.1]), with no significant difference in AA risk. In girls, no increased asthma risk was observed in Q1Q4, but AA risk was significantly higher in Q4Q4 (adjusted OR: 1.78 [95% CI: 1.21–2.6]).

**Conclusion:**

Our results demonstrated that BMI changes during early childhood impact asthma risks, particularly that the risk of NA in boys increases with BMI changes during early childhood, and the risk of AA in girls increases with consistently high BMI.

## INTRODUCTION

The number of people with asthma in the world was estimated to be 262 million in 2019.^1^ Asthma is a heterogeneous disease, usually characterized by chronic airway inflammation.^2^ Researchers have explored asthma classifications, including phenotypes (classification based on various clinical symptoms) and endotypes (classification based on complex pathophysiological mechanisms), to understand its mechanisms, make a prognosis, and tailor treatments.^3^, ^4^ Numerous studies have identified various risk factors for the development of asthma, among which obesity has emerged as a significant contributor.^5^ Changes in body mass index (BMI) and weight during early childhood have also been identified as a risk factor for childhood asthma.^6^, ^7^, ^8^ Emerging evidence indicates variations in susceptibility and the impact of risk factors, such as personal and family history, race, and birth weight between sexes.^9^, ^10^ However, there are still few reports on the relationship between BMI changes during early childhood and asthma prevalence divided by each phenotype and sex, and the limited results are inconsistent.^8^, ^11^, ^12^ The present study aimed to examine the impact of BMI changes during early childhood on school‐age asthma prevalence classified by phenotypes and sex based on a nationwide longitudinal survey in Japan.

## METHODS

### Study participants

We analyzed data from the Longitudinal Survey of Infants in the 21st Century, an ongoing national cohort study conducted by the Japanese Ministry of Health, Labour, and Welfare. The study enrolled infants born in Japan between January 10 and 17 or July 10 and 17, 2001 and followed them up with questionnaires. A total of 47,015 questionnaires were returned by the 53,575 families mailed the first questionnaire when the infants were 6 months old (88% response rate). Follow‐up questionnaires were sent annually to those participating. Vital statistics were linked to each child, with details on sex, singleton/multiple birth status, gestational age, length, weight, and parental age when delivered.

### Children's changes in BMI status

We focused on changes in the BMI status between BMI at birth and at the age of 7 years. We first calculated BMI at birth and at the age of 7 years by dividing the weight in kilograms by the square of the height in meters and divided the participants into quartile categories at both ages. We then created the following categories by comparing BMI quartiles they belonged to at both ages: Q1Q1 (i.e., Q1 at birth and Q1 at age 7), Q1Q4 (i.e., Q1 at birth and Q4 at age 7), Q4Q1 (i.e., Q4 at birth and Q1 at age 7), Q4Q4 (i.e., Q4 at birth and Q4 at age 7), and others. The reference group was comprised of other children. We obtained weight and height information at birth from vital statistics and those at the age of 7 years from the self‐administered questionnaire when the children were 7 years old. Of the 47,015 children, we excluded 152 children who did not have BMI information at birth as well as 14,747 children who did not have BMI information at the age of 7 years, resulting in 32,116 children being included in the analysis.

### Hospital visits due to bronchial asthma from 7 to 8 years of age

To examine the impact of changes in the BMI status on BA prevalence, we used the children's history of hospital visits due to BA from 7 to 8 years of age as an indicator of the health status. The survey asked whether the child had seen a doctor at least once in the previous 1 year for several common illnesses. For the present study, we examined whether there was at least one hospital consultation for BA from 7 to 8 years of age. We also categorized BA into allergic asthma (AA) and nonallergic asthma (NA) according to the presence or absence of hospital visits due to atopic dermatitis, allergic rhinitis, and food allergy. We defined AA as BA visits with at least one visit due to these allergic conditions, and NA as BA visits with none of these visits. This information on hospital visits from 7 to 8 years of age was obtained from the follow‐up questionnaire at the age of 8 years (i.e., at the eighth follow‐up survey), which excluded 1587 children due to being lost to follow‐up, leaving 30,529 children for the final analysis.

### Statistical analysis

After conducting descriptive analyses by the BMI change category, we first examined the association of BMI quartiles at 7 years of age with hospital visits due to BA, AA, or NA from 7 to 8 years of age. We subsequently examined the association of the BMI change category between birth and 7 years of age with hospital visits due to BA, AA, or NA from 7 to 8 years of age as the main analysis.

For all analyses, we used a logistic regression model and first estimated the crude odds ratio (OR) and 95% confidence interval (CI) (crude model), adjusted first for children's factors as well as parental factors (fully adjusted model). We selected potential confounders based on previous studies or prior knowledge of associations between the BMI status and BA.

Children's factors included: sex (dichotomous), singleton birth status (dichotomous), preterm birth (dichotomous: gestational age ≥37 vs. <37 weeks), parity (dichotomous: 0 vs. ≥ 1 birth), breastfeeding status at infancy (categorical: formula feeding, partial breastfeeding, and exclusive breastfeeding), and daycare attendance (dichotomous). Parental factors included maternal age at delivery (continuous), maternal smoking status [categorical: nonsmoker, light smoker (≤10 cigarettes/day), and heavy smoker (more than 10 cigarettes per day)], parental educational attainment (categorical), and residential area (categorical: wards, cities, and towns or villages). The children's sex, singleton birth status, gestational age, and parity, and maternal age at delivery were listed in the birth records. The breastfeeding status and maternal smoking status were obtained from the first survey, when the children were 6 months old. Daycare attendance and parental educational attainment, used as an indicator of the socioeconomic status, were obtained from the second survey, when the children were 18 months old. We reclassified the original eight educational categories into four: university (4 years) or higher, junior college, high school, and less than high school. Residential information was obtained from the national census conducted in 2000.

All CIs were calculated at the 95% level, and STATA SE version 18 (Statacorp, College Station, TX, USA) was used for all analyses. This study was approved by the Institutional Review Board of Okayama University Graduate School of Medicine, Dentistry, and Pharmaceutical Sciences (No. 1506‐073).

## RESULTS

### Demographic characteristics of eligible children

The mean BMI and SD for each category at the age of 7 were as follows: Q1 (13.6 ± 0.71), Q2 (14.8 ± 0.25), Q3 (15.7 ± 0.31), and Q4 (17.9 ± 1.72).

Table 1 shows demographic characteristics of the 32,116 eligible children. Five BMI‐change categories are shown: Q1Q1 (*N* = 2344), Q1Q4 (*N* = 1667), Q4Q1 (*N* = 1642), Q4Q4 (*N* = 2415), and reference (*N* = 24,048). Children in the Q1Q1 and Q1Q4 categories had a higher incidence of preterm birth.

**TABLE 1:** Demographic characteristics of eligible children (*n* = 32,116).

	BMI change category				
	Reference (*N* = 24,048)	Q1Q1 (*N* = 2344)	Q1Q4 (*N* = 1667)	Q4Q1 (*N* = 1642)	Q4Q4 (*N* = 2415)
Characteristics of children					
Sex, *n* (%)^a^					
Boys	12,573 (52.5)	1157 (47.4)	935 (55.5)	744 (45.6)	1260 (52.5)
Girls	11,385 (47.5)	1284 (52.6)	749 (44.5)	889 (54.4)	1140 (47.5)
Singleton or multiple birth, *n* (%)^a^					
Singleton	23,534 (98.2)	2327 (95.3)	1617 (96)	1610 (98.6)	2383 (99.3)
Multiple birth	424 (1.8)	114 (4.7)	67 (4)	23 (1.4)	17 (0.7)
Mean gestational age, weeks (SD)^a^	38.9 (1.5)	38.5 (2.1)	38.6 (1.8)	39.1 (1.5)	39.1 (1.3)
Term birth, *n* (%)^a^	22,939 (95.8)	2230 (91.4)	1546 (91.8)	1580 (96.8)	2336 (97.3)
Preterm birth, *n* (%)^a^	1019 (4.3)	211 (8.6)	138 (8.2)	53 (3.3)	64 (2.7)
Parity, *n* (%)^a^					
0	11,809 (49.3)	1385 (56.7)	926 (55)	714 (43.7)	1019 (42.5)
≥1	12,149 (50.7)	1056 (43.3)	758 (45)	919 (56.3)	1381 (57.5)
Breastfeeding status^b^					
Formula feeding	1278 (5.4)	150 (6.2)	115 (6.9)	100 (6.2)	141 (5.9)
Partial breastfeeding	17,099 (71.8)	1820 (74.9)	1218 (72.8)	1171 (72.2)	1721 (72.3)
Exclusive breastfeeding at 6–7 months of age	5432 (22.8)	459 (18.9)	340 (20.3)	350 (21.6)	517 (21.7)
Daycare attendance, *n* (%)^c^					
Not attend	20,027 (84.8)	2035 (84.8)	1364 (82.4)	1377 (85.3)	1934 (81.7)
Attend	3587 (15.2)	365 (15.2)	291 (17.6)	237 (14.7)	434 (18.3)
Parental characteristics					
Mean maternal age at delivery, years (SD)^a^	30.4 (4.3)	30.2 (4.4)	30.4 (4.4)	30.5 (4.3)	30.7 (4.3)
Maternal smoking status, *n* (%)^b^					
Nonsmoker	20,710 (86.8)	2104 (86.5)	1430 (85.3)	1417 (87.1)	1988 (83.2)
Smoker (≤10 cigarettes/day)	2184 (9.2)	227 (9.3)	150 (8.9)	124 (7.6)	251 (10.5)
Smoker (more than 10 cigarettes/day)	970 (4.1)	101 (4.2)	97 (5.8)	86 (5.3)	150 (6.3)
Maternal educational attainment, *n* (%)^c^					
University or higher	3714 (15.8)	382 (16)	249 (15.1)	230 (14.3)	313 (13.2)
Junior college or vocational school	10,190 (43.3)	1032 (43.3)	703 (42.6)	707 (43.9)	980 (41.5)
High school	8734 (37.1)	884 (37.1)	615 (37.3)	585 (36.4)	952 (40.3)
Junior high school and others	890 (3.8)	88 (3.7)	83 (5)	87 (5.4)	119 (5)
Paternal educational attainment, *n* (%)^c^					
University or higher	9388 (40.2)	924 (39)	597 (36.5)	603 (37.8)	814 (34.8)
Junior college or vocational school	3652 (15.6)	372 (15.7)	259 (15.8)	270 (16.9)	395 (16.9)
High school	8770 (37.6)	894 (37.8)	673 (41.2)	605 (37.9)	958 (40.9)
Junior high school and others	1534 (6.6)	178 (7.5)	106 (6.5)	118 (7.4)	174 (7.4)
Residential area, *n* (%)					
Wards	5368 (22.4)	604 (24.7)	327 (19.4)	371 (22.7)	504 (21)
Cities	14,118 (58.9)	1433 (58.7)	1006 (59.7)	1001 (61.3)	1362 (56.8)
Towns or villages	4472 (18.7)	404 (16.6)	351 (20.8)	261 (16)	534 (22.3)

Abbreviation: BMI, body mass index.

^a^ Obtained from the birth record.

^b^ Obtained from the first survey (at the age of 6 months).

^c^ Obtained from the second survey (at the age of 18 months).

### Relationship between BMI change and asthma phenotypes, divided by sex

In Q4 at the age of 7–8, risks of BA and AA were significantly higher (adjusted OR: 1.22 [95% CI: 1.06–1.39], 1.43 [95% CI: 1.17–1.73]), whereas no significant difference was observed in the risk of NA (1.16 [95% CI: 0.96–1.39]; Table 2).

**TABLE 2:** Crude and adjusted^a^ ORs for associations between BMI at 7 years of age and hospital visit due to bronchial asthma from 7 to 8 years of age.

	*Ncase*/*N*	% of cases	Crude ORs (95% CI)	Adjusted^a^ ORs 95% CI)
Bronchial asthma				
Q1	436/7632	5.7	1 (ref.)	1 (ref.)
Q2	451/7628	5.9	1.04 (0.91–1.19)	1.01 (0.88–1.16)
Q3	479/7663	6.3	1.1 (0.96–1.26)	1.08 (0.94–1.23)
Q4	533/7606	7.0	1.24 (1.09–1.42)	1.22 (1.06–1.39)
Allergic asthma				
Q1	203/7399	2.7	1.07 (0.87–1.3)	1.11 (0.9–1.36)
Q2	190/7367	2.6	1 (ref.)	1 (ref.)
Q3	216/7400	2.9	1.14 (0.93–1.38)	1.14 (0.93–1.39)
Q4	259/7332	3.5	1.38 (1.14–1.67)	1.43 (1.17–1.73)
Nonallergic asthma				
Q1	233/7429	3.1	1 (ref.)	1 (ref.)
Q2	261/7438	3.5	1.12 (0.94–1.34)	1.1 (0.92–1.33)
Q3	263/7447	3.5	1.13 (0.95–1.35)	1.12 (0.93–1.35)
Q4	274/7347	3.7	1.2 (1–1.43)	1.16 (0.96–1.39)

Abbreviations: BMI, body mass index; CI, confidence interval; OR, odds ratio.

^a^ Adjusted for child factors (sex, singleton or not, preterm birth, parity, breastfeeding status, and daycare attendance), parental factors (maternal age at delivery, maternal smoking status, maternal educational attainment, and paternal educational attainment), and residential area.

In Q1Q4, risks of BA, AA, and NA were significantly increased (adjusted OR: 1.4 [95% CI: 1.16–1.7], 1.4 [95% CI: 1.06–1.85], and 1.41 [95% CI: 1.1–1.8], respectively). In Q4Q4, only the risk of AA was significantly higher (adjusted OR: 1.29 [95% CI: 1.01–1.65]), and not that of BA or NA (Table 3). In boys of Q1Q4, only the risks of BA and NA were significantly higher (adjusted OR: 1.47 [95% CI: 1.17–1.85] and 1.56 [95% CI: 1.16–2.1], respectively), and not that of AA (1.37 [95% CI: 0.98–1.92]; Table 4). In girls of Q1Q4, there was no significant increase in the risk of BA, AA, or NA. In Q4Q4 girls, the risk of AA was significantly higher (adjusted OR: 1.78 [95% CI: 1.21–2.6]). This trend was not observed in boys.

**TABLE 3:** Crude and adjusted^a^ ORs for associations between BMI change and hospital visit due to bronchial asthma from 7 to 8 years of age.

	N*case*/*N*	% of cases	Crude ORs (95% CI)	Adjusted^a^ ORs (95% CI)
Bronchial asthma				
Reference	1388/22,796	6.1	1 (ref.)	1 (ref.)
Q1Q1	140/2315	6.0	0.99 (0.83–1.19)	0.99 (0.83–1.2)
Q1Q4	137/1614	8.5	1.43 (1.19–1.72)	1.4 (1.16–1.7)
Q4Q1	86/1537	5.6	0.91 (0.73–1.14)	0.97 (0.77–1.22)
Q4Q4	148/2267	6.5	1.08 (0.9–1.28)	1.1 (0.92–1.32)
Allergic asthma				
Reference	619/22,027	2.8	1 (ref.)	1 (ref.)
Q1Q1	68/2243	3.0	1.08 (0.84–1.39)	1.11 (0.85–1.44)
Q1Q4	59/1536	3.8	1.38 (1.05–1.81)	1.4 (1.06–1.85)
Q4Q1	46/1497	3.1	1.1 (0.81–1.49)	1.19 (0.87–1.61)
Q4Q4	76/2195	3.5	1.24 (0.97–1.58)	1.29 (1.01–1.65)
Nonallergic asthma				
Reference	769/22,177	3.5	1 (ref.)	1 (ref.)
Q1Q1	72/2247	3.2	0.92 (0.72–1.18)	0.91 (0.7–1.17)
Q1Q4	78/1555	5.0	1.47 (1.16–1.87)	1.41 (1.1–1.8)
Q4Q1	40/1491	2.7	0.77 (0.56–1.06)	0.8 (0.57–1.1)
Q4Q4	72/2191	3.3	0.95 (0.74–1.21)	0.95 (0.74–1.22)

Abbreviations: BMI, body mass index; CI, confidence interval; OR, odds ratio.

^a^ Adjusted for child factors (sex, singleton or not, preterm birth, parity, breastfeeding status, and daycare attendance), parental factors (maternal age at delivery, maternal smoking status, maternal educational attainment, and paternal educational attainment), and residential area.

**TABLE 4:** Adjusted^a^ ORs for associations between BMI change and hospital visit due to bronchial asthma from 7 to 8 years of age, divided by sex.

	Adjusted^a^ ORs (95% CI)	
	Boys	Girls
Bronchial asthma		
Reference	1 (ref.)	1 (ref.)
Q1Q1	1 (0.79–1.28)	0.98 (0.73–1.32)
Q1Q4	1.47 (1.17–1.85)	1.27 (0.91–1.78)
Q4Q1	0.87 (0.64–1.19)	1.11 (0.8–1.55)
Q4Q4	1.02 (0.81–1.28)	1.26 (0.94–1.67)
Allergic asthma		
Reference	1 (ref.)	1 (ref.)
Q1Q1	0.99 (0.7–1.4)	1.32 (0.88–1.97)
Q1Q4	1.37 (0.98–1.92)	1.46 (0.89–2.39)
Q4Q1	1.07 (0.71–1.61)	1.39 (0.87–2.22)
Q4Q4	1.07 (0.78–1.48)	1.78 (1.21–2.6)
Nonallergic asthma		
Reference	1 (ref.)	1 (ref.)
Q1Q1	1.01 (0.74–1.4)	0.76 (0.5–1.16)
Q1Q4	1.56 (1.16–2.1)	1.14 (0.73–1.8)
Q4Q1	0.69 (0.43–1.1)	0.92 (0.58–1.46)
Q4Q4	0.98 (0.71–1.34)	0.9 (0.59–1.38)

Abbreviations: BMI, body mass index; CI, confidence interval; OR, odds ratio.

^a^ Adjusted for child factors (singleton or not, preterm birth, parity, breastfeeding status, and daycare attendance), parental factors (maternal age at delivery, maternal smoking status, maternal educational attainment, and paternal educational attainment), and residential area.

## DISCUSSION

We examined the relationship between BMI changes during early childhood and school‐age asthma prevalence classified by phenotypes and sex with a nationwide longitudinal survey. The results suggested that there was a sex difference in the impact of BMI changes during early childhood on asthma risks. Overall, Q1Q4 had significantly higher risks of BA, AA, and NA. In boys, BA and NA risks were significantly higher in Q1Q4, but no significant difference was observed in AA risks. In girls, there was no significant difference in the risk of BA, AA, or NA in Q1Q4. The AA risk was significantly higher in Q4Q4 only in girls.

Q1Q4 had significantly higher risks of BA. This finding is consistent with previous papers showing that BMI changes during early childhood are a risk factor for asthma.^6^, ^7^, ^8^ When divided by subtypes and sex, there were findings that agreed with past reports and others that did not. In a cohort study from Taiwan, the risk of nonatopic asthma, but not atopic asthma, was significantly higher in a group with rapid weight gain from birth to 18 months. The risk of nonatopic asthma was also significantly higher in boys, but no significant difference was observed in girls.^12^ On the other hand, there was a study with conflicting results. A French cohort study found a significant positive correlation with an increased risk of AA, but not NA, in a group with marked increases in BMI from birth to 9–11 years of age. The risk of AA was significantly higher in boys, but not girls. It Is Important to consider known asthma risks to examine the BMI–asthma relationship, and preterm birth is one of them.^13^ In preterm children, a higher standardized weight‐for‐age during catch‐up growth may decrease the risk of asthma/recurrent wheeze.^14^ Fat distribution differences between races were suggested.^15^ The relationship between body fat and respiratory outcomes varies depending on the distribution.^16^ Racial differences and whether preterm birth is taken into account may cause the differences in results. This study performed larger‐scale verification in a Japanese population while taking into account preterm birth and showed that BMI changes during early childhood were positively correlated with the risk of asthma, especially NA in boys.

This relationship may be mediated by the timing of adiposity rebound (AR), which typically occurs between 5 and 7 years of age. Early AR is a strong predictor of future obesity and metabolic syndrome.^17^ Rapid weight gain between 1 and 5 years old was shown to be a clinical feature of early AR.^18^ As a previous study, which provided evidence that obesity‐associated adipose tissue dysfunction developed in early childhood,^19^ fat accumulation in early timing may start the metabolic dysregulation. These may lead to impaired lung development, the mechanical effect of central adiposity, and elevated levels of cytokines associated with airway inflammation and remodeling.^20^ Structural mechanical change is one of the hypotheses regarding the adiposity‐associated asthma mechanism. Overweightness and obesity in children were positively correlated with dysanapsis (physiological incongruence between the growth of lung parenchyma and caliber of the airways, abnormal FEV_1_/FVC despite normal FEV_1_ and FVC). Dysanapsis was shown to be correlated with the risk of acute exacerbation of severe asthma and increased risk of systemic steroid administration.^21^ Dysanaptic lung growth was already observed in healthy infants, and it was correlated with weight gain and higher BMI.^22^ There is also a research which showed that lower FEV_1_/FVC in healthy boys than girls of school age.^23^ This combination of dysanaptic lung growth and boy characteristics could lead to the increased risk of NA in boys.

It should be noted that the risk of AA was significantly higher in girls in Q4Q4, whereas this trend was not observed in boys. It is also worth noting the increased risk of AA without BMI changes during early childhood. A French cohort study also reported that the risk of atopic asthma was significantly higher in girls with high BMI values both at birth and school age, but not in boys.^8^ Some studies have reported contradictory results. A Swedish cohort study showed that persistently high BMI during childhood increased the risk of doctor‐diagnosed asthma at school age, and there was no sex difference.^24^ Previous cross‐sectional studies showed that there were no or negative associations between BMI and FE_NO_ in children.^16^, ^25^ The mechanism to explain why this tendency was observed only in females remains unclear. In this previous study, children aged 7–8 years were targeted, and the effect of sex hormones was expected to be small. Further investigation is needed not only regarding BMI changes but also consistently high BMI.

There have been no previous studies of this scale in Japan that have described the relationship between BMI changes during early childhood and increased risk of asthma in school‐age children. This data further emphasized the importance of growth management during early childhood. Given that the results differed depending on sex and phenotype classification, we hope that future research will be designed to further advance the analysis of the mechanisms of childhood asthma.

A major strength of our study was the utilization of large‐scale, nationwide birth cohort data from Japan, contributing to a diverse and representative sample. This enhances the generalizability and validity of our findings. We also benefited from high response rates to our questionnaires, reducing the potential for response bias and improving the reliability of results.

However, our study has a few limitations. First, the accuracy of diagnosing asthma and related conditions relied on questionnaire data, which could have led to underestimations. While diagnoses are generally reliable given Japan's accessible healthcare system, inconsistencies may occur as they depend on individual physicians' assessments rather than a standardized protocol. Second, despite adjusting for known asthma risk factors, some data, such as maternal BMI during pregnancy and family history of allergic diseases, were missing. Third, with no date specified for the skin‐prick test or allergen‐specific IgE, we used history of allergic diseases within 1 year to categorize asthma into allergic and nonallergic. While a previous study found an association between sensitization patterns and allergic disease,^26^ allergy testing is essential for the accurate classification of allergic and nonallergic asthma.^27^ According to the classification criteria in this study, patients who were IgE positive but did not have atopic dermatitis, allergic rhinitis, or food allergy may have been misclassified as NA instead of AA. In fact, the proportion of AA to NA was small compared to previous studies.^8^, ^12^, ^27^ Thus, there is a risk of misclassifying patients into allergic and nonallergic asthma subgroups. This misclassification bias may have led to an underestimation of the effect estimates. We need to consider the classification difference when comparing with previous studies. These factors should be included in future studies to enhance understanding. Fourth, 14,747 out of 47,015 children were excluded due to missing BMI information, which may have introduced selection bias. The excluded population was more likely to be maternal smoking and have lower socioeconomic status, which could have raised their risk of asthma.^28^ This bias may have resulted in an underestimation of the effect estimates.

## CONCLUSION

Our results demonstrated that BMI changes during early childhood impact asthma risks, particularly that the risk of NA in boys increases with BMI changes during early childhood, and the risk of AA in girls increases with consistently high BMI. These findings highlight the importance of monitoring BMI changes during early childhood and considering sex differences when assessing asthma risks.

## AUTHOR CONTRIBUTIONS

Yabuuchi, T. contributed to the conception, interpretation of data, and drafting the manuscript; Ikeda, M. contributed to the interpretation of data and revision of the manuscript; Matsumoto, N. contributed to the revision of the manuscript; Tsuge, M. contributed to the revision of the manuscript; Yorifuji, T. contributed to obtaining the data, the study design, data analysis, and revision of the manuscript; Tsukahara, H. contributed to comprehensive supervision and revision of the manuscript. All authors read and approved the final manuscript.

## CONFLICT OF INTEREST STATEMENT

The authors declare no conflicts of interest.

## ETHICS STATEMENT

This study was approved by the Institutional Review Board of Okayama University Graduate School of Medicine, Dentistry and Pharmaceutical Sciences (No. 1506‐073). The study received ethical approval.

## References

[1] Global burden of 369 diseases and injuries in 204 countries and territories, 1990–2019: a systematic analysis for the global burden of disease study 2019. Lancet. 2020;396(10258):1204–1222. 33069326 10.1016/S0140-6736(20)30925-9 PMC7567026

[2] Reddel HK , Bacharier LB , Bateman ED , Brightling CE , Brusselle GG , Buhl R , et al. Global initiative for asthma strategy 2021: executive summary and rationale for key changes. Eur Respir J. 2022;59(1):2102730. 34667060 10.1183/13993003.02730-2021 PMC8719459

[3] Conrad LA , Cabana MD , Rastogi D . Defining pediatric asthma: phenotypes to endotypes and beyond. Pediatr Res. 2021;90(1):45–51. 33173175 10.1038/s41390-020-01231-6 PMC8107196

[4] Conrad LA , Cabana MD , Rastogi D , Just J , Just J , Bourgoin‐Heck M , et al. Clinical phenotypes in asthma during childhood. Clin Exp Allergy. 2017;47(1):848–855. 28422351 10.1111/cea.12939

[5] Deng X , Ma J , Yuan Y , Zhang Z , Niu W . Association between overweight or obesity and the risk for childhood asthma and wheeze: an updated meta‐analysis on 18 articles and 73,252 children. Pediatr Obes. 2019;14(9):e12532. 31033249 10.1111/ijpo.12532

[6] Rzehak P , Wijga AH , Keil T , Eller E , Bindslev‐Jensen C , Smit HA , et al. Body mass index trajectory classes and incident asthma in childhood: results from 8 European Birth Cohorts‐‐a Global Allergy and Asthma European Network initiative. J Allergy Clin Immunol. 2013;131(6):1528–1536. 10.1016/j.jaci.2013.01.001 23403049

[7] van der Sonnenschein‐ Voort AMM , Howe LD , Granell R , Duijts L , Sterne JAC , Tilling K , et al. Influence of childhood growth on asthma and lung function in adolescence. J Allergy Clin Immunol. 2015;135(6):1435–1443.e7. 25577593 10.1016/j.jaci.2014.10.046 PMC4452091

[8] Chastang J , Baiz N , Parnet L , Cadwallader JS , De Blay F , Caillaud D , et al. Changes in body mass index during childhood and risk of various asthma phenotypes: a retrospective analysis. Pediatr Allergy Immunol. 2017;28(3):273–279. 28140475 10.1111/pai.12699

[9] Tse SM , Rifas‐Shiman SL , Coull BA , Litonjua AA , Oken E , Gold DR . Sex‐specific risk factors for childhood wheeze and longitudinal phenotypes of wheeze. J Allergy Clin Immunol. 2016;138(6):1561–1568.e6. 27246527 10.1016/j.jaci.2016.04.005 PMC5083247

[10] Tse SM , Coull BA , Sordillo JE , Datta S , Gold DR . Gender‐ and age‐specific risk factors for wheeze from birth through adolescence. Pediatr Pulmonol. 2015;50(10):955–962. 25348842 10.1002/ppul.23113 PMC4800823

[11] Tsai H‐J , Wang G , Hong X , Yao T‐C , Ji Y , Radovick S , et al. Early life weight gain and development of childhood asthma in a prospective birth cohort. Ann Am Thorac Soc. 2018;15(10):1197–1204. 29979628 10.1513/AnnalsATS.201712-921OC PMC6321993

[12] Ho C‐H , Gau C‐C , Lee W‐F , Fang H , Lin C‐H , Chu C‐H , et al. Early‐life weight gain is associated with non‐atopic asthma in childhood. World Allergy Organ J. 2022;15(8):100672. 35983567 10.1016/j.waojou.2022.100672 PMC9356168

[13] van der Sonnenschein‐ Voort AMM , Arends LR , de Jongste JC , Annesi‐Maesano I , Arshad SH , Barros H , et al. Preterm birth, infant weight gain, and childhood asthma risk: a meta‐analysis of 147,000 European children. J Allergy Clin Immunol. 2014;133(5):1317–1329. 10.1016/j.jaci.2013.12.1082 24529685 PMC4024198

[14] Shah J , Shadid ILC , Carey VJ , Laranjo N , O'Connor GT , Zeiger RS , et al. Early‐life weight status and risk of childhood asthma or recurrent wheeze in preterm and term offspring. J Allergy Clin Immunol Pract. 2023;11(7):2125–2132.e1. 37088369 10.1016/j.jaip.2023.03.059 PMC10330365

[15] He Q , Horlick M , Thornton J , Wang J , Pierson RNJ , Heshka S , et al. Sex and race differences in fat distribution among Asian, African‐American, and Caucasian prepubertal children. J Clin Endocrinol Metab. 2002;87(5):2164–2170. 10.1210/jcem.87.5.8452 11994359

[16] den Dekker HT , Ros KPI , de Jongste JC , Reiss IK , Jaddoe VW , Duijts L . Body fat mass distribution and interrupter resistance, fractional exhaled nitric oxide, and asthma at school‐age. J Allergy Clin Immunol. 2017;139(3):810–818.e6. 27592177 10.1016/j.jaci.2016.06.022

[17] Rolland‐Cachera MF , Deheeger M , Bellisle F , Sempé M , Guilloud‐Bataille M , Patois E . Adiposity rebound in children: a simple indicator for predicting obesity. Am J Clin Nutr. 1984;39(1):129–135. 6691287 10.1093/ajcn/39.1.129

[18] Koyama S , Ichikawa G , Kojima M , Shimura N , Sairenchi T , Arisaka O . Adiposity rebound and the development of metabolic syndrome. Pediatrics. 2014;133(1):e114–e119. 24366997 10.1542/peds.2013-0966

[19] Landgraf K , Rockstroh D , Wagner IV , Weise S , Tauscher R , Schwartze JT , et al. Evidence of early alterations in adipose tissue biology and function and its association with obesity‐related inflammation and insulin resistance in children. Diabetes. 2014;64(4):1249–1261. 10.2337/db14-0744 25392242

[20] Vijayakanthi N , Greally JM , Rastogi D . Pediatric obesity‐related asthma: the role of metabolic dysregulation. Pediatrics. 2016;137(5):e20150812. 27244776 10.1542/peds.2015-0812 PMC4845863

[21] Forno E , Weiner DJ , Mullen J , Sawicki G , Kurland G , Han YY , et al. Obesity and airway Dysanapsis in children with and without asthma. Am J Respir Crit Care Med. 2017;195(3):314–323. 27552676 10.1164/rccm.201605-1039OC PMC5328183

[22] Sanchez‐Solis M , Forno E , Morales E , Garcia‐Marcos L . Role of body mass index in unbalanced (dysanaptic) lung growth of healthy infants. Pediatr Pulmonol. 2024;59(11):2939–2946. 10.1002/ppul.27161 38967254

[23] Dockery DW , Berkey CS , Ware JH , Speizer FE , Ferris BGJ . Distribution of forced vital capacity and forced expiratory volume in one second in children 6 to 11 years of age. Am Rev Respir Dis. 1983;128(3):405–412. 6614634 10.1164/arrd.1983.128.3.405

[24] Loid P , Goksör E , Alm B , Pettersson R , Möllborg P , Erdes L , et al. A persistently high body mass index increases the risk of atopic asthma at school age. Acta Paediatr. 2015;104(7):707–712. 25818987 10.1111/apa.13015 PMC4654247

[25] Yao T‐C , Tsai H‐J , Chang S‐W , Chung R‐H , Hsu J‐Y , Tsai M‐H , et al. Obesity disproportionately impacts lung volumes, airflow and exhaled nitric oxide in children. PLoS One. 2017;12(4):e0174691. 28376119 10.1371/journal.pone.0174691 PMC5380337

[26] Lee E , Suh DI , Lee S‐Y , Jung S , Yoon SJ , Cho H , et al. Association between sensitization and allergic diseases in 7‐years‐old Korean children. Asian Pac J Allergy Immunol. 2019;39(4):231–240. 10.12932/AP-201218-0464 31310150

[27] Sinisgalli S , Collins MS , Schramm CM . Clinical features cannot distinguish allergic from non‐allergic asthma in children. J Asthma. 2012;49(1):51–56. 22136286 10.3109/02770903.2011.631244

[28] Kadowaki T , Matsumoto N , Suzuki E , Mitsuhashi T , Takao S , Yorifuji T . Breastfeeding at 6 months of age had a positive impact on overweight and obesity in Japanese adolescents at 15 years of age. Acta Paediatr. 2023;112(1):106–114. 36168735 10.1111/apa.16551

